# Differences between co-cultures and monocultures in testing the toxicity of particulate matter derived from log wood and pellet combustion

**DOI:** 10.1371/journal.pone.0192453

**Published:** 2018-02-21

**Authors:** Stefanie Kasurinen, Mikko S. Happo, Teemu J. Rönkkö, Jürgen Orasche, Jorma Jokiniemi, Miika Kortelainen, Jarkko Tissari, Ralf Zimmermann, Maija-Riitta Hirvonen, Pasi I. Jalava

**Affiliations:** 1 Department of Environmental and Biological Sciences, University of Eastern Finland, Kuopio, Finland; 2 HICE-Helmholtz Virtual Institute of Complex Molecular Systems in Environmental Health-Aerosols and Health, Munich, Germany; 3 Cooperation Group Comprehensive Molecular Analytics, Helmholtz Zentrum München, German Research Center for Environmental Health, Munich, Germany; 4 Institute of Chemistry, University of Rostock, Rostock, Germany; Utah State University, UNITED STATES

## Abstract

**Background:**

*In vitro* studies with monocultures of human alveolar cells shed deeper knowledge on the cellular mechanisms by which particulate matter (PM) causes toxicity, but cannot account for mitigating or aggravating effects of cell-cell interactions on PM toxicity.

**Methods:**

We assessed inflammation, oxidative stress as well as cytotoxic and genotoxic effects induced by PM from the combustion of different types of wood logs and softwood pellets in three cell culture setups: two monocultures of either human macrophage-like cells or human alveolar epithelial cells, and a co-culture of these two cell lines. The adverse effects of the PM samples were compared between these setups.

**Results:**

We detected clear differences in the endpoints between the mono- and co-cultures. Inflammatory responses were more diverse in the macrophage monoculture and the co-culture compared to the epithelial cells where only an increase of IL-8 was detected. The production of reactive oxygen species was the highest in epithelial cells and macrophages seemed to have protective effects against oxidative stress from the PM samples. With no metabolically active cells at the highest doses, the cytotoxic effects of the PM samples from the wood log combustion were far more pronounced in the macrophages and the co-culture than in the epithelial cells. All samples caused DNA damage in macrophages, whereas only beech and spruce log combustion samples caused DNA damage in epithelial cells. The organic content of the samples was mainly associated with cytotoxicity and DNA damage, while the metal content of the samples correlated with the induction of inflammatory responses.

**Conclusions:**

All of the tested PM samples induce adverse effects and the chemical composition of the samples determines which pathway of toxicity is induced. *In vitro* testing of the toxicity of combustion-derived PM in monocultures of one cell line, however, is inadequate to account for all the possible pathways of toxicity.

## Introduction

According to the Global Burden of Disease Study, air pollution and especially particulate matter (PM) emissions from the combustion of solid fuels are a leading cause of mortality and morbidity worldwide [1]. While there is sufficient evidence available to classify PM emissions from coal combustion as carcinogenic by the International Agency for Research on Cancer [2], data about the PM emissions from wood combustion is still insufficient and thus wood smoke has been classified as a probable carcinogen (group 2A) [2]. Most of the mortality caused by wood smoke is due to cooking on open fires in developing countries [3]. However, recently it has been shown that wood smoke has the same adverse health effects also in the developed world [4,5]. Regulating agencies like the Environmental Protection Agency (EPA) or the European Commission often assume that all PM of a certain size range is equally as dangerous [6,7]. Nonetheless, many studies indicate that the adverse effects are greater if particles are composed e.g. of carbonaceous compounds and/or have a high metal content compared to particles, which are mainly composed of inorganic soluble salts [8,9,10,11,12].

The adverse health effects of PM emissions from wood combustion have been studied with monocultures of several cell types [13], but also co-cultures of two or more cell types have been applied [14]. Each of these systems has advantages and disadvantages. For example, A549 cells, which are commonly used to represent type II pulmonary cells, contribute to an immune response mainly by chemokine secretion, even though inflammation is thought to be the main mechanism by which combustion-derived PM causes adverse health effects [15]. In contrast, cultures of immune cells can overestimate the extent of the immune response. Independent of the combustion conditions, wood combustion yields mainly ultrafine and fine PM with a cut-off diameter of less than 1 μm [16]. These particles deposit deep into the alveolar cavities [17], where mechanical clearing systems are virtually non-existent and clearance is largely depending on cells of the host’s immune system.

In a previous study [18] we determined that the chemical composition of PM emissions from the combustion of three different wood logs varies considerably, as do the adverse effects of the samples in A549 epithelial cells. This indicates that combustion-derived PM of different sources may exhibit different toxic effects, depending on the chemical composition and physical properties. To evaluate more accurately, how these wood combustion PM samples affect cell metabolism and if cell-cell interactions could have mitigating or aggravating effects on PM toxicity, we tested them in two more *in vitro* systems. The first system consisted of a monoculture of differentiated THP-1 cells to represent immune cells. The second system was a simple co-culture of A549 cells and THP-1 cells to estimate the effects of cell-cell interaction on PM toxicity. The co-culture setup used in the present study is easily reproducible and represents cell-cell interactions better than monocultures. However, even with the most intricate *in vitro* systems it is not possible to mimic and determine the overall systemic effects of PM exposure. Thus, *in vivo* experiments would add to the complete evaluation of the PM from heating appliances. From the immune cells and the co-culture, we determined two parameters of cell viability, the cellular metabolic activity (CMA) and the membrane integrity of the cells and the induction of oxidative stress in all three set-ups. We also tested the ability of the samples to induce inflammatory responses, and the ability of the samples to cause DNA damage. These two new experimental setups in combination with our previously published results [18] allow to draw more precise conclusions, how the particles affect cellular metabolism and if the seen responses are cell-type specific or if the particles elicit general adverse effects independent of the cell type.

## Methods

### Sample collection and preparation for toxicological analyses

The collection and preparation of the PM samples used in this study have been described in detail previously [18,19]. Briefly, we collected particulate emissions with an aerodynamic diameter below 1 μm (PM_1_) from the combustion of three different wood logs (birch, beech and spruce) in a modern masonry heater and PM_1_ emissions from the combustion of spruce pellets in a fully automated pellet boiler. PM_1_ samples were collected after the porous tube and first ejector diluter on polyfluoroethylene (PTFE) substrates using a Dekati^®^ gravimetric impactor (DGI), with an air flow of 70 liters/min. The DGI separates PM according to their aerodynamic diameter (*d*_*a*_) into four size fractions: (1) PM_0.2_ (*d*_*a*_ < 0.2 μm), (2) PM_0.2–0.5_ (*d*_*a*_ = 0.2–0.5 μm), (3) PM_0.5–1_ (*d*_*a*_ = 0.5–1.0 μm), (4) PM_1-2.5_ (*d*_*a*_ = 1.0–2.5 xm), (5) PM_> 2.5_ (*d*_*a*_ > 2.5 μm)[20]. Fraction 1–3 were pooled to yield the PM_1_ emissions of the appliances. We used methanol to extract the PM_1_ samples for the chemical and toxicological analyses. The exact extraction procedure is described in detail in [21,22], briefly PTFE filters were quartered aseptically and immersed in 30 ml HPLC-grade methanol in glass tubes. The tubes were placed in a sonicator bath and sonicated for 30 min at RT. The extracts were pooled in a 1 l round-bottom flask and the extraction was repeated. After the second extraction PTFE filters were dried and extraction efficiencies (> 90%) were calculated. The methanol was evaporated to a final volume of 5–10 ml using a rotary evaporator with 150 mbar and 32°C PM samples were then distributed on a mass basis in Kimax^®^ glass tubes and the final methanol residue was evaporated using N_2_-gas. Samples were stored at -20°C until chemical or toxicological analysis. We analyzed the concentration of the chemical elements Al, Ca, Cu, Fe, K, Mg, Na, Ni, Pb, V, and Zn in the collected and extracted PM_1_ samples by inductively coupled-plasma mass spectrometry (ICP-MS, Agilent Technologies 7700, USA), as well as the concentration of the ions Cl^-^, NO_3_^-^, SO_4_^2-^, and PO_4_^3-^ by ion chromatography (IC, Metrohm, Switzerland). Details of these procedures are described previously in [18] and [21]. Analyses of the organic, elemental and polycyclic aromatic hydrocarbon (PAH) content of the PM_1_ samples are described in [18]. Briefly, we analyzed the content of organic and elemental carbon in the samples with a thermal-optical carbon analyser (Lab OC-EC Aerosol Analyzer, Sunset Laboratory Inc. USA) according to the NIOSH 5040 manual. The concentration of PAH compounds, alkylated PAH compounds and oxygenated PAH compounds was determined by gas chromatography-mass spectrometry (GC-MS, 6890N GC-5973 INERT MSD, Agilent Technologies, CA; or Shimadzu GCMS-QP2010 Ultra, Shimadzu, Japan, equipped with a 60 m BPX-5 column 0.22 mm ID, 0.25 μm film, SGE, Australia). We did not analyze the endotoxin content of the PM_1_ samples because it is very unlikely that there is a significant amount of endotoxin in primary PM_1_ emissions. For the toxicological analyses, PM_1_ samples were dispersed in 10% dimethyl sulfoxide (DMSO) in Embryo-transfer water at a concentration of 5 mg/ml. Complete dispersion was ensured by sonication for 30 minutes in a sonicator bath.

### Cell culture and study design

The culturing and exposure conditions for A549 cells have been previously described in detail in [18]. In brief, A549 human alveolar epithelial cells were purchased from ATCC (ATCC^®^ CCL-185^™^) and maintained in Dulbecco’s Modified Eagle Medium (DMEM, Sigma-Aldrich, USA), supplemented with 10% fetal bovine serum (FBS), 2 mM L-glutamine (L-glut) and 100 U/ml penicillin/streptomycin (pen/strep) (all Sigma-Aldrich, USA). For the exposure experiments, cells were seeded at a density of 150,000 cells/ml/well in 12-well plates. This seeding density corresponds to cell densities between 400,000 and 600,000 cells/ml and end of the 24 h exposure period.

THP-1 human monocytes cells (DSMZ ACC 16) were purchased from the German Collection of Microorganisms and Cell Cultures (DSMZ, Germany). THP-1 cells were maintained in Roswell Park Memorial Institute (RPMI) 1640 culture medium (Life Sciences, Gibco) supplemented with 10% FBS, 2 mM L-glut and 100 U/ml pen/strep (all Sigma-Aldrich, USA). Since THP-1 cells are routinely maintained in suspension, cells were differentiated into active macrophage-like cells with phorbol 12-myristate 13-acetate (PMA) before the experiments. Between 15.000.000 and 20.000.000 cells from a maintenance flask were centrifuged and resuspended in 10 ml culture medium containing 0.5 μg/ml PMA. This cell suspension was seeded in a new tissue culture flask and the cells were incubated at 37°C and 5% CO_2_ in a humidified incubator for 90 minutes. This is enough time for the cells to attach loosely to the bottom of the flask. We tested PMA concentrations ranging from 5 ng/ml to 1 μg/ml and incubation times ranging from 1 h to 48 h and found that our chosen protocol yields the most consistent results in the shortest time frame. After the 90-minute incubation, floating cells were aspirated and the attached cells were detached by rinsing them with Dulbecco’s phosphate buffered saline (PBS). Cells were washed twice with 10 ml PBS before counting them. We seeded THP-1 macrophages at 300.000/ml/well in 12-well plates. This seeding density yields approximately 400,000 cells/ml at the end of the 24 h exposure period. Activation and differentiation of the THP-1 cells into mature macrophages was determined by flow cytotometric staining with an Alexa488-labelled antibody (life technologies, USA) against cluster of differentiation 14 (CD14), which is expressed only in mature macrophages, not in monocytic precursor cells [23].

For the co-culture experiments, we first seeded 120.000 A549 cells in the 12-well-plates and let them attach for 4 h before seeding 24.000 differentiated and washed THP-1 cells on top of them. These co-cultured cells were then incubated at 37°C and 5% CO_2_ for 40 hours in a humidified incubator before the exposure to the PM samples. Seeding densities for the A549/THP-1 co-culture were chosen so that the cell density at the end of the 24 h exposure period was approximately 400,000–600,000 cells/ml.

The exposure protocol was identical for the three cell culture setups. One hour prior to the exposure of the cells with the PM_1_ samples, the culture medium was replaced and the PM samples were prepared. Cells were exposed to four increasing particulate concentrations (25 μg/ml, 75 μg/ml, 150 μg/ml and 200 μg/ml) in duplicates for 24 hours at 37°C and 5% CO_2_ in a humidified incubator. A variety of positive and negative exposure agents were included in the assay setup, e.g. lipopolysaccharide (LPS) for inflammation, methyl methanesulfonate (MMS) for genotoxicity as well as solvent and blank controls. After the 24-hour exposure period, the cell culture medium was salvaged and frozen at -80°C for the cytokine analyses. The cells were then detached by adding 1 ml trypsin-EDTA and incubating them for 5 minutes at 37°C; after this 0.1 ml FBS was added to stop trypsin activity. Two 100 μl aliquots of the duplicates were analyzed for the cellular metabolic activity (CMA) by (3-(4,5-dimethylthiazol-2-yl)-2,5-diphenyltetrazolium bromide) MTT-assay; a 200 μl aliquot of the duplicates was analyzed for the membrane integrity of the cells by propidium iodide (PI) exclusion assay and the intracellular production of reactive oxygen species (ROS). After this, the procedure diverged for the mono- and co-cultures. The cells of the monocultures were used in the single-cell gel electrophoresis (SCGE) assay and the remaining cells of the co-cultures were frozen at -80 degrees for further analyses.

### Inflammatory parameters

The pro-inflammatory markers tumor necrosis factor alpha (TNFα), interleukin 6 (IL-6) and interleukin 8 (IL-8) from the monocultures, as well as IL-8 and transforming growth factor beta (TGFβ) from the co-cultured cells were determined by sandwich enzyme-linked immunosorbent assay (ELISA, R&D-Systems, USA) according to the manufacturer’s instructions.

The pro-inflammatory cytokines and chemokines granulocyte macrophage colony-stimulating factor (GM-CSF), interferon gamma (IFNγ), interleukin 1 beta (IL-1β), interleukin 10 (IL-10), TNFα, IL-6, as well as the vascular endothelial growth factor alpha (VEGFα) were determined from the supernatant of the A549/THP-1 co-cultures with the U-PLEX kit from Meso Scale Diagnostics on a Sector 2400A imager Reader. The binding of the capture antibodies and the coating of the plates was conducted for 1 h at room temperature in accordance with the manufacturer’s instructions.

### Oxidative stress

For the analysis of cellular ROS production, we used a 200 μl aliquot from both duplicates. First the cells were centrifuged, the supernatant was discarded and the cells were resuspended in 220 μl PBS without FBS, because FBS interferes with ROS measurement. Then 2 x 100 μl aliquots were plated in 96-well-plates and 8 μl of 2',7'-dichlorodihydrofluorescein diacetate (H_2_DCF-DA) (0.5 μM in dimethyl sulfoxide (DMSO)) was added. 2',7'-dichlorofluorescein (DCF) fluorescence was measured immediately and at 30 minutes and 60 minutes (485 nm excitation and 530 nm emission, VICTOR3^™^ Multilabel Counter model 1420–051, PerkinElmer, USA). We then plotted the fluoresence units against the time and calculated the area under the curve (AUC) for each well. Then, the values were normalized by dividing the AUC for exposed cells by the AUC of unexposed control cells.

### Cytotoxicty

#### Membrane permeability

After the ROS measurement, we added 7.2 μl PI-solution (0.5 mg/ml in PBS) to the wells and incubated the cells at 37°C and 5% CO_2_ in a humidified incubator for 20 minutes. After this incubation the baseline PI fluorescence was assessed (540 nm excitation and 610 nm emission, VICTOR3^™^ Multilabel Counter model 1420–051, PerkinElmer, USA) and we added 20 μl of a Triton X-100 lysing solution (10% Triton-X-100 in ddH_2_O) to the wells and incubated the cells a second time for 20 minutes at room temperature. Then, the maximum PI fluorescence was measured as before and we calculated the viability of the cells according to the following formula: percentage viability = 100 –((PI_baseline_/PI_max_)*100).

#### Cellular metabolic activity

For analysis of the CMA we used the MTT-assay. We distributed two 100 μl aliquots of the cell suspension of each of the duplicates in a 96-well-plate and immediately added 25 μl MTT-solution (5 mg/ml in PBS). The cells were then incubated for two hours at 37°C and 5% CO_2_ in a humidified incubator for two hours, during which metabolically active cells take up the yellow MTT and convert it into a purple formazan. After this incubation, we added 100 μl sodium dodecyl sulfate (SDS)-lysing buffer (0.2 g/ml SDS in 50% (v/v) ddH2O, 50% (v/v) dimethylformamide) and lysed the cells for 16 h at 37°C to extract all the formazan into solution before measuring absorbance at 570 nm in a multiplate-reader (VICTOR3^™^ Multilabel Counter model 1420–051, PerkinElmer, USA).

### Genotoxicity

The alkaline version of the SCGE assay was used in this study to determine DNA damage. Two 20 μl cell aliquots were suspended in 70 μl low melting point agarose (0.5% in PBS) and plated on 75 mm microscope slides. Thereafter, cells were lysed at +4°C for 1 h in lysis buffer (2.5 M NaCl, 0.1 M Na_2_-EDTA, 0.01 M Trizma^®^-Base, 1% Triton X-100, pH 10). The DNA was unwound by immersing the slides for 40 min in electrophoresis buffer (0.3 N NaOH, 1 mM Na_2_-EDTA, pH > 13) in the dark in the electrophoresis tank. We then applied 24 V/300 mA for 20 min to separate intact from fragmented DNA. After the electrophoresis, the slides were rinsed three times with neutralizing buffer (0.4 M Tris(hydroxymethyl)aminomethane, pH 7.5) and dried with 100% ethanol. DNA was stained with ethidium bromide and comets were analysed with Comet assay IV software (Perceptive Instruments Ltd., UK). The amount of DNA in the tail was used as the parameter to estimate DNA damage, since it produces results, which can be more easily compared between laboratories [24].

### Statistical analysis and correlation of the chemical composition of the samples with the measured toxicity endpoints

Levene’s test for equality of variances was conducted before analysis of variance (ANOVA) of the data. We used Dunnett’s test to assess significance between the magnitude of responses of unexposed control cells and cells exposed to particulate samples. Tukey’s test was used to assess signifcance of the responses between the PM samples at the same PM dose, and also to determine significance of the responses between different setups (monocultures and co-culture) of the same sample. If equal distribution of the samples could not be assumed (p < 0.05 in Levene’s), we used Welch’s F-test with Dunnett’s T3 posthoc test to assess significance between samples or cell culture setups. The non-parametric Kruskal-Wallis test was used to assess significant differences between samples and control and also between samples in the SCGE assay because of the lower number of replicates. Significance was met at *p* < 0.05 for all analyses conducted in this study.

The analysis of the correlation between the chemical composition of the samples and the toxicological responses was carried out using Spearman’s two-tailed rank correlation. We included all PM_1_ doses. We considered correlation coefficients significant at *p* < 0.05. IBM SPSS statistics software, version 21 was used for all statistical analyses.

## Results

### Inflammation

The inflammatory responses of the A549 monoculture after exposure of the cells to the PM_1_ samples are described in detail in [18]. Briefly, only IL-8 levels were increased after exposure to the PM_1_ samples, no increase in either TNFα or IL-6 could be detected.

**Fig 1:**
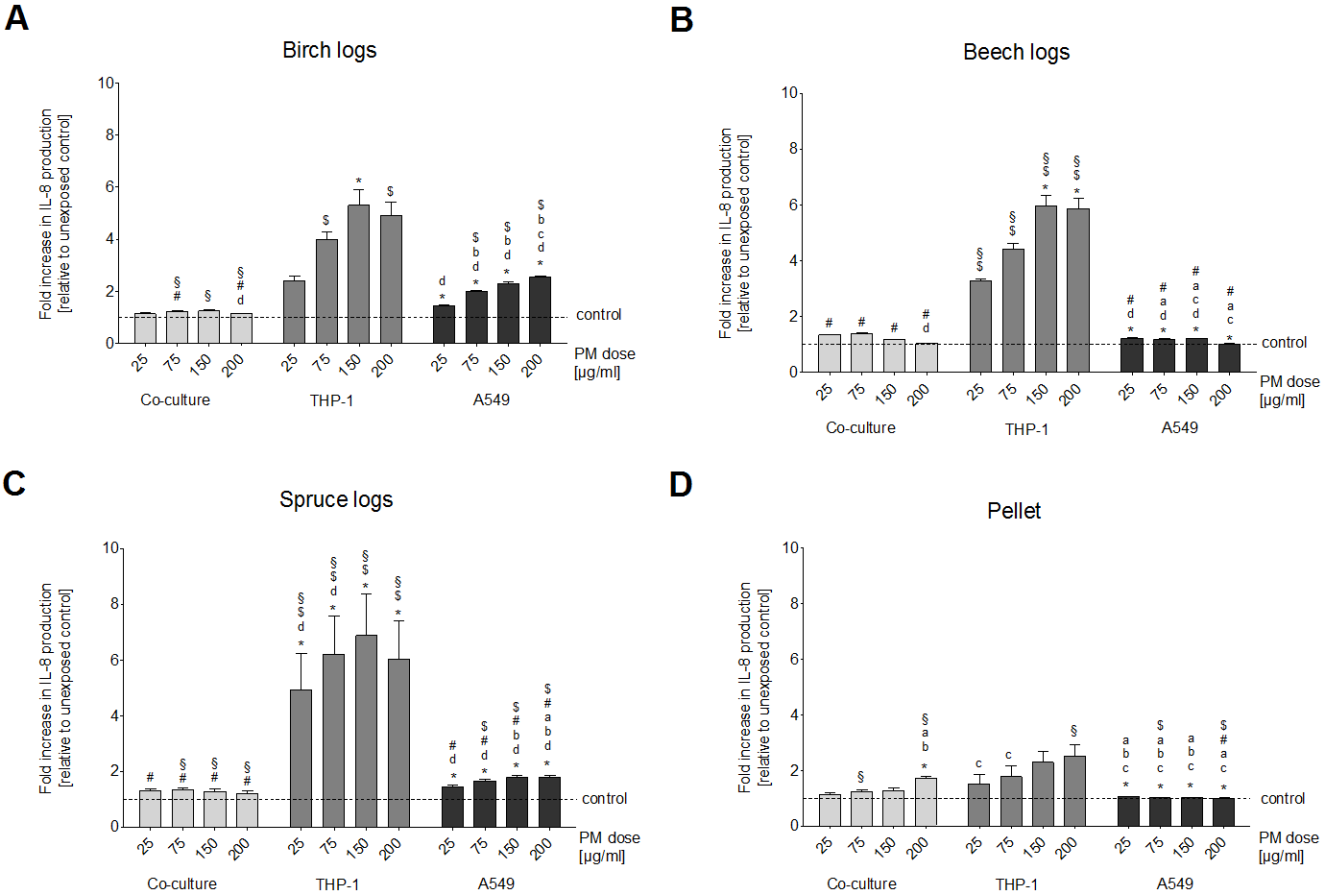
IL-8 production. Production of the pro-inflammatory marker IL-8 after a 24 h exposure of three different cell culture setups (A549 and THP-1 monocultures and A549/THP-1 co-culture) to four doses (25, 75, 150 and 200 μg/ml) of PM_1_ samples from the combustion of three different wood logs and wood pellets. Bars represent the increase in IL8 secretion by the cells in comparison to unexposed control cells + SEM of the experimental averages. Asterisks indicate significance from blank control. **a** indicates significance from the birch log PM_1_ sample, **b** indicates significance from the beech log PM_1_ sample, **c** indicates significance from the spruce log PM_1_ sample, **d** indicates significance from the pellet combustion PM_1_ sample; **§** indicates significance from the A549 monoculture, **#** indicates significance from the THP-1 monoculture, **$** indicates significance from the A549/THP-1 co-culture.

All three logwood combustion samples caused an increase in IL-8 inTHP-1 cells that was approximately twice as high as that of cells exposed to the pellet combustion sample (Fig 1A). However, due to the high cytotoxicity of the log wood combustion samples, the IL-8 production of the THP-1 cells reached a plateau at PM doses of 150 μg/ml and 200 μg/ml (Fig 1) with IL-8 concentrations of approximately 500 pg/ml for the birch PM_1_ sample and between 600 and 700 pg/ml for the beech and spruce PM_1_ samples. The absolute level of IL-8 produced by unexposed control cells was lower in THP-1 cells (approximately 100 pg/ml) than that of the A549 cells (approximately 230 pg/ml)[18], thus the fold-increase compared to unexposed control cells was significantly higher in THP-1 cells than in A549 cells for all three log wood combustion samples (Fig 1A, 1B and 1C).

The TNFα concentration of the THP-1 cells followed a similar pattern as the IL-8 concentration (S1 Fig). The largest amount of TNFα secretion was induced by the birch and beech log combustion samples, and for these samples we also detected the plateau at 150 μg/ml and 200 μg/ml (S1 Fig). Interestingly, the PM_1_ sample from the pellet combustion caused an increase in the TNFα production of THP-1 cells which was almost as high as for the log wood combustion samples. The increase of TNFα concentration for this sample was dose dependent and significance was met at PM doses of 150 μg/ml and 200 μg/ml (S1 Fig).

The relative increase of IL-8 concentration in the co-cultured cells was the lowest of the three tested *in vitro* setups (Fig 1). However, it has to be taken into consideration that the IL-8 concentration in unexposed control cells varied substantially between setups, with 100 pg/ml for THP-1 cells, 230 pg/ml for A549 cells and 47 ng/ml for the co-culture.

**Fig 2:**
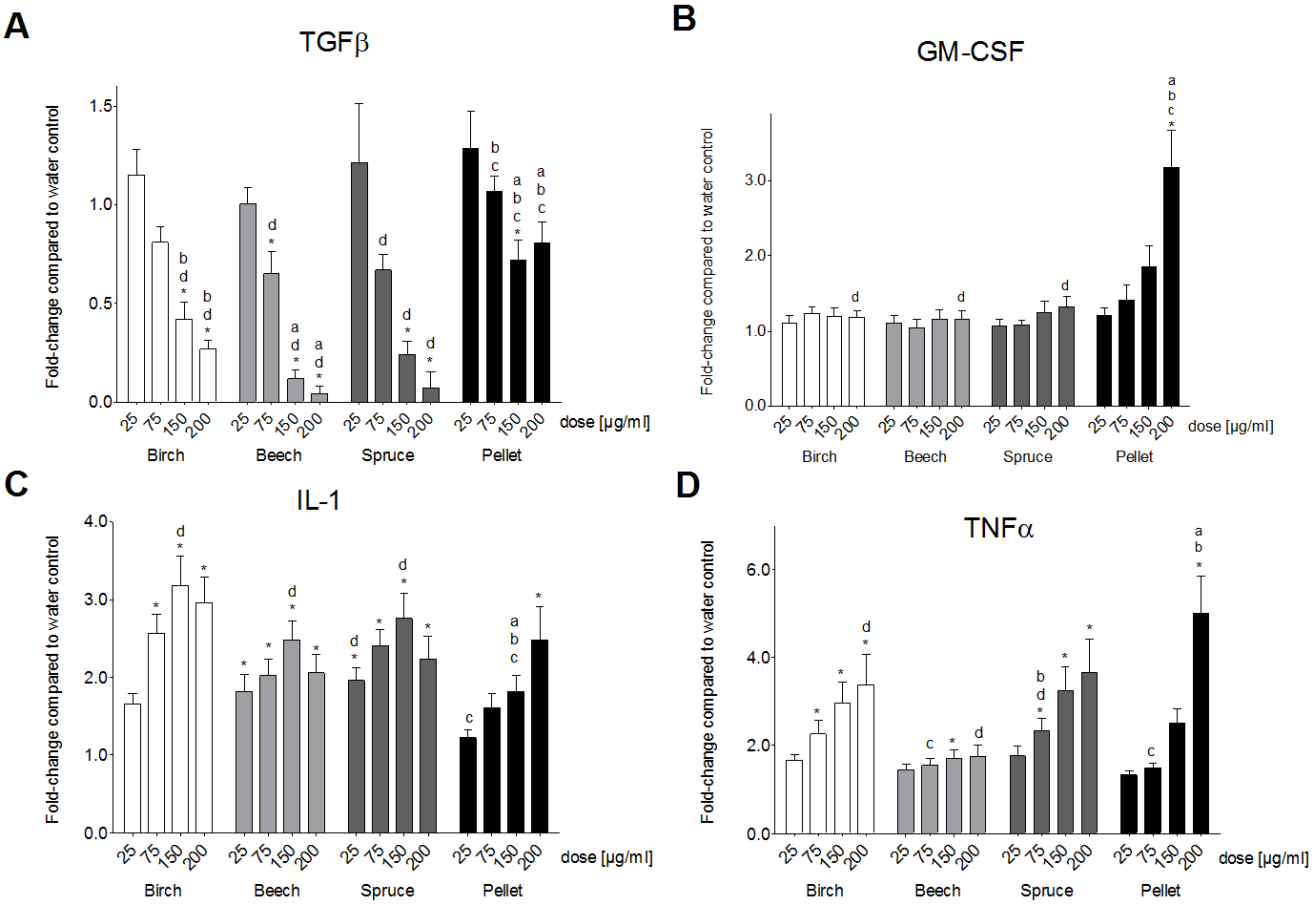
Inflammation. Production of the pro-inflammatory markers (**A**) TGFβ, (**B**) GM-CSF, (**C**) IL-1β and (**D**) TNFα by a co-culture of A549 and THP-1 cells after a 24 h exposure of the cells to four doses (25, 75, 150 and 200 μg/ml) of PM_1_ samples from the combustion of three different wood logs and wood pellets. Bars represent the fold-change compared to unexposed control cells + SEM of the experimental averages. Asterisks indicate significance from unexposed control cells. **a** indicates significance from the birch log PM_1_ sample, **b** indicates significance from the beech log PM_1_ sample, **c** indicates significance from the spruce log PM_1_ sample, **d** indicates significance from the pellet combustion PM_1_ sample.

We detected significant increases of TNFα production in the co-culture (Fig 2). For the birch and beech PM_1_ samples, the relative increase in TNFα was higher in the THP-1 monoculture than in the co-culture, whereas for the spruce and pellet PM_1_ samples the increase was higher in the co-cultures than in the THP-1 monoculture (Fig 1, S1 Fig). The PM_1_ sample from the beech log combustion did not cause a dose-dependent increase in the co-culture of A549 and THP-1 cells (Fig 2D). All other samples caused a dose-dependent increase of TNFα concentration with significance from unexposed control cells at PM doses of 75 μg/ml and higher for the birch and spruce log combustion samples and 200 μg/ml for the pellet combustion sample (Fig 2D).

We measured altogether nine inflammatory parameters from the cell culture supernatants of the A549/THP-1 co-culture, but accurately quantifiable results were obtained only for TGFβ, GM-CSF, IL-1β and TNFα. While we detected an increase of the concentrations of the latter three markers upon exposure of the cells to the PM_1_ samples, the concentration of TGFβ decreased dose-dependently as compared to unexposed control cells (Fig 2A). The decrease was the most severe for the PM_1_ sample from the beech log combustion and the least severe for the PM_1_ sample from the pellet combustion (Fig 2A). Contrarily, the PM_1_ sample from the pellet combustion caused the highest increase in GM-CSF production, while the log wood combustion samples caused no significant changes as compared to unexposed control cells (Fig 2B). IL-1β concentration was increased for all four PM_1_ samples (Fig 2C). The log wood combustion samples caused significant increases of IL-1β at PM concentrations of 75 μg/ml and higher, but the IL-1β concentration after exposure of the cells to 200 μg/ml was lower than the IL-1β concentration after exposure of the cells to 150 μg/ml (Fig 2C). In contrast, the pellet combustion PM_1_ sample caused a dose-dependent increase of IL-1β, but significance from unexposed control cells was met only at 200 μg/ml (Fig 2C).

### Oxidative stress

**Fig 3:**
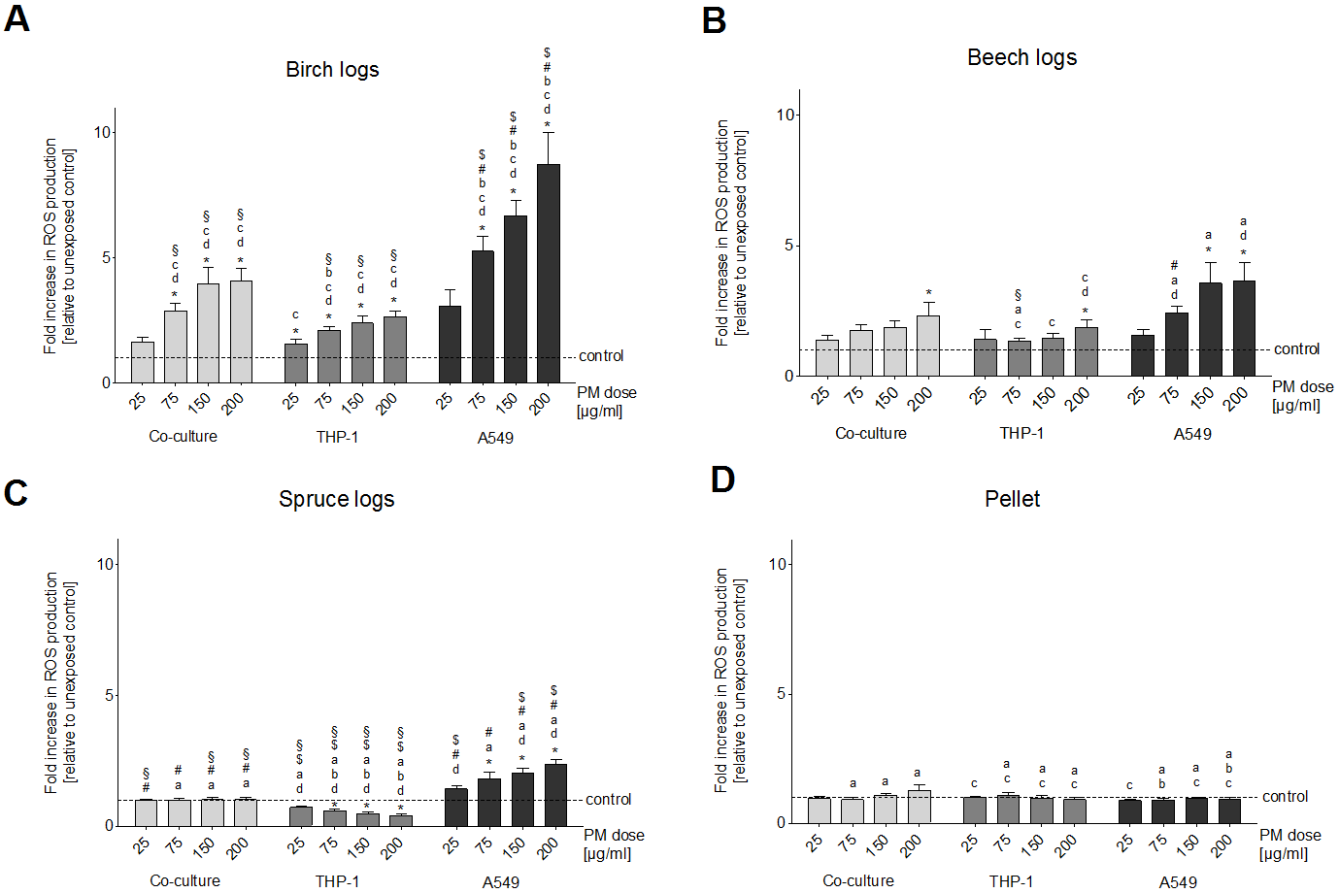
Oxidative stress. Oxidative stress assessed with the DCF-assay after a 24 h exposure of three different cell culture setups (A549 and THP-1 monocultures and A549/THP-1 co-culture) to four doses (25, 75, 150 and 200 μg/ml) of particulate samples from the combustion of three different wood logs and wood pellets. Each bar represents the average of eight experiments. Whiskers indicate the standard error of the mean (SEM), asterisks indicate significance from blank control. **a** indicates significance from the birch log PM_1_ sample, **b** indicates significance from the beech log PM_1_ sample, **c** indicates significance from the spruce log PM_1_ sample, **d** indicates significance from the pellet combustion PM_1_ sample. **§** indicates significance from the A549 monoculture, **#** indicates significance from the THP-1 monoculture, **$** indicates significance from the A549/THP-1 co-culture.

Most of the samples did not induce an increase in intracellular ROS in THP-1 cells (Fig 3). The only sample that caused a dose-dependent and significant increase was the PM_1_ sample from the birch log combustion (Fig 3A). The PM_1_ sample from the spruce log combustion caused a significant decrease of ROS production in THP-1 cells compared to unexposed control cells (Fig 3C).

This result is in stark contrast with the results obtained in A549 monocultures we have published earlier [18]. An increase in the amount of ROS in A549 cells was seen for all three log wood combustion PM_1_ samples (Fig 3A–3C). The intracellular concentration of ROS in A549 cells was significantly higher than that of THP-1 cells and co-cultured cells for the birch and spruce log combustion samples (Fig 3A and 3C), but no such significance was found for the beech log combustion sample (Fig 3B). The PM_1_ sample from the pellet combustion did not cause significant differences between the ROS production in the three cell culture setups (Fig 3D).

Only the PM_1_ sample from the birch log combustion that caused an increase of intracellular ROS in the A549/THP-1 co-culture (Fig 3A). We did not find significant differences in the level of ROS between the THP-1 monocultures and the co-cultured cells for the birch and beech log combustion samples or the pellet combustion sample (Fig 3A, 3B and 3D). For the PM_1_ sample from the spruce log combustion we detected significant differences between these two cell culture setups at all PM concentrations (Fig 3C).

### Cytotoxicity

#### Cellular metabolic activity

**Fig 4:**
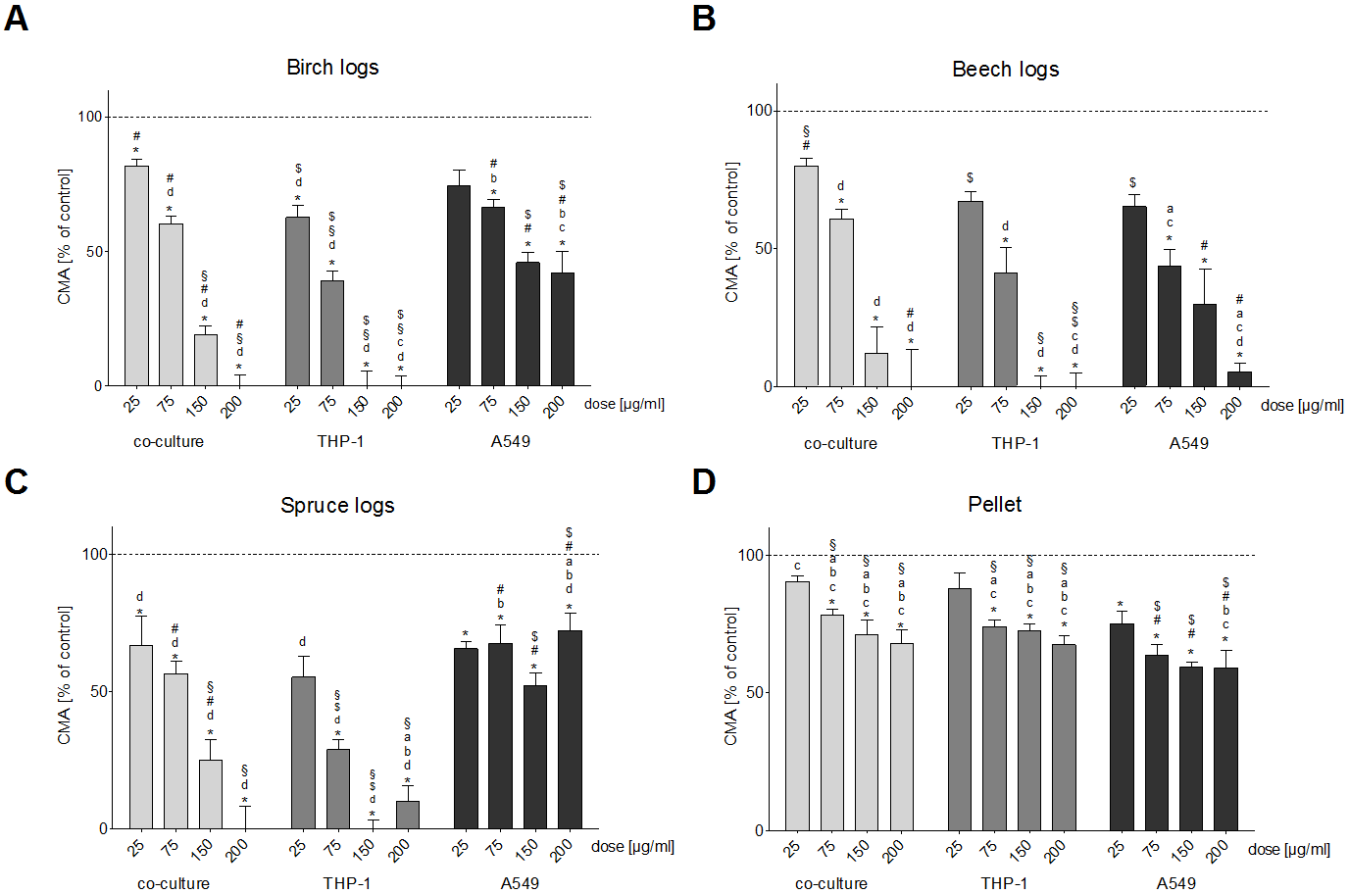
CMA. Cellular Metabolic Activity assessed with the MTT-test after a 24 h exposure of three different cell culture setups (A549 and THP-1 monocultures and A549/THP-1 co-culture) to four doses (25, 75, 150 and 200 μg/ml) of particulate samples from the combustion of three different wood logs and wood pellets. Each bar represents the average of eight experiments. Whiskers indicate the standard error of the mean (SEM), asterisks indicate significance from unexposed control cells. **a** indicates significance from the birch log PM_1_ sample, b indicates significance from the beech log PM_1_ sample, **c** indicates significance from the spruce log PM_1_ sample, **d** indicates significance from the pellet combustion PM_1_ sample. **§** indicates significance from the A549 monoculture, **#** indicates significance from the THP-1 monoculture, **$** indicates significance from the A549/THP-1 co-culture.

The PM_1_ samples from the birch, beech and spruce log combustion induced a dose-dependent decrease of the CMA of THP-1 cells; in fact, we found no or only a minimal amount of metabolically active cells at PM concentrations of 150 and 200 μg/ml (Fig 4A). For the spruce log combustion PM_1_ sample, the decrease of CMA in the THP-1 monoculture was significantly more severe than the decrease of CMA in A549 cells at PM doses of 75 μg/ml and higher, and more severe than the CMA decrease of the co-cultured cells at 200 μg/ml (Fig 4C). The PM_1_ sample from the pellet combustion caused the least amount of CMA damage. The decrease of CMA was significant at PM_1_ doses of 75 μg/ml, 150 μg/ml and 200 μg/ml for THP-1 cells (Fig 4D).

In A549 cells, the birch log combustion PM_1_ did not cause as severe a reduction of the CMA than in THP-1 cells (Fig 4A): at PM doses of 150 μg/ml and 200 μg/ml CMA was approximately 45% compared to 0% for THP-1 cells. However, the decrease of CMA caused by the PM_1_ sample from the beech log combustion in A549 cells was more severe (Fig 4B). The decrease of CMA in A549 cells after exposure to the PM_1_ sample from the spruce log combustion did not follow dose-dependency, but it was significantly lower than that of unexposed control cells at all PM concentrations (Fig 4C).

In the co-culture of A549 and THP-1 cells, severity of CMA reduction was on approximately the same level as for the THP-1 monoculture. For the PM_1_ sample from the birch log combustion the decrease was 80%, 60% and 20% for particulate concentration of 25 μg/ml, 75 μg/ml and 150 μg/ml; and at a PM dose of 200 μg/ml no metabolically active cells were found (Fig 4A). The PM1 sample from the beech log combustion caused similar decreases of CMA in this cell model (Fig 4B). The decrease of CMA after exposure to the PM_1_ sample from the spruce log combustion was dose-dependent and significant from unexposed control cells (Fig 4C).

The PM_1_ sample from the pellet combustion caused the least amount of CMA in all cell culture models. Compared to unexposed control cells the decrease of CMA was significant at PM_1_ doses of 75 μg/ml, 150 μg/ml and 200 μg/ml for the co-culture and THP-1 cells and at all tested PM_1_ concentrations for the A549 cells (Fig 4D).

#### PI exclusion

**Fig 5:**
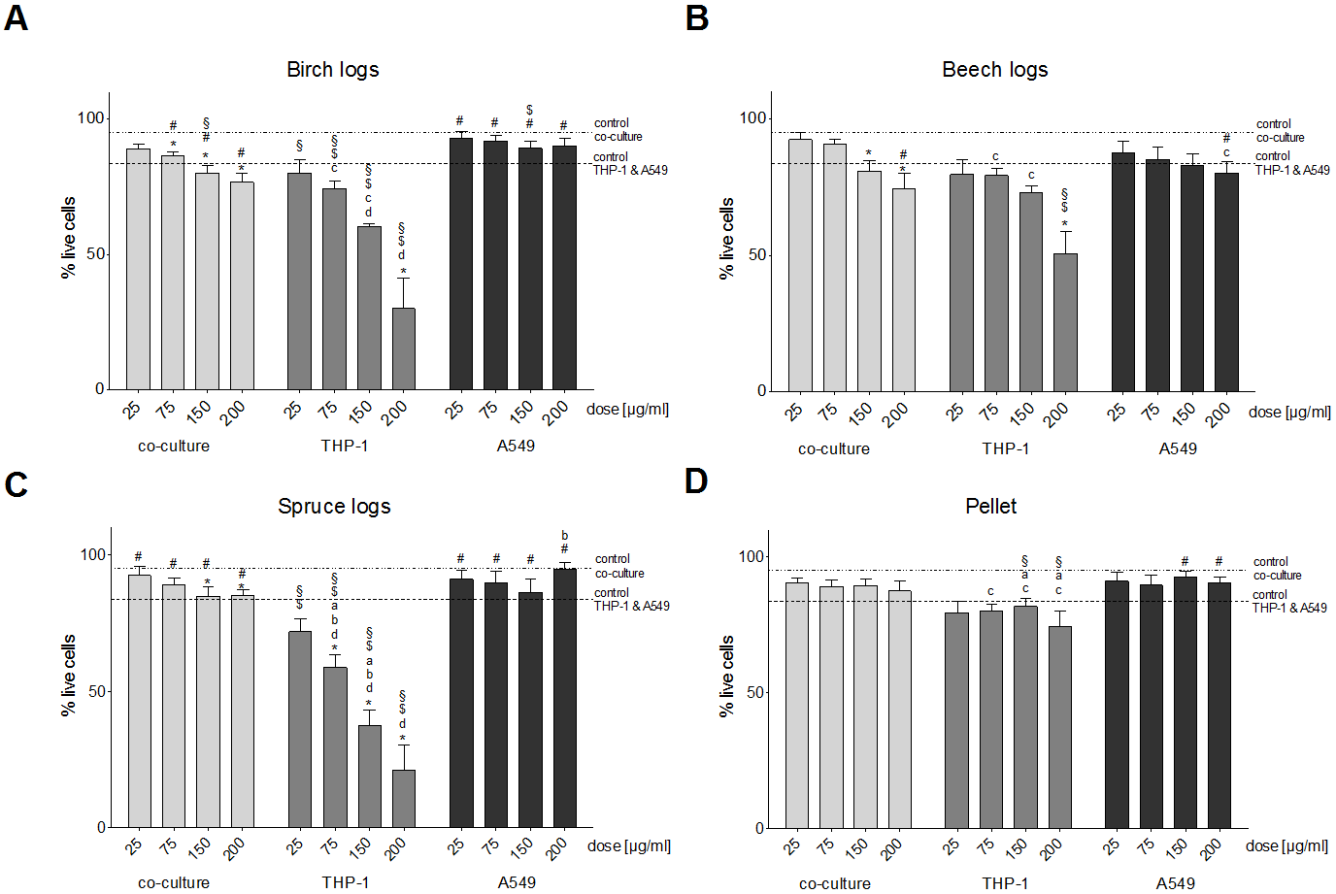
Cell membrane integrity. Cell membrane integrity assessed with the PI exclusion assay after a 24 h exposure of three different cell culture setups (A549 and THP-1 monocultures and A549/THP-1 co-culture) to four doses (25, 75, 150 and 200 μg/ml) of particulate samples from the combustion of three different wood logs and wood pellets. Each bar represents the average of eight experiments. Whiskers indicate the standard error of the mean (SEM), asterisks indicate significance from unexposed control cells. **a** indicates significance from the birch log PM_1_ sample, **b** indicates significance from the beech log PM_1_ sample, **c** indicates significance from the spruce log PM_1_ sample, **d** indicates significance from the pellet combustion PM_1_ sample. **§** indicates significance from the A549 monoculture, **#** indicates significance from the THP-1 monoculture, **$** indicates significance from the A549/THP-1 co-culture.

The results from the THP-1 monoculture regarding reduced cell membrane integrity from the log wood combustions differed from the results in the A549 monoculture [18] and the A549/THP-1 co-culture for the PM_1_ samples (Fig 5). All three samples induced a dose-dependent decrease of cell membrane integrity (Fig 5A–5C); however, the severity varied between samples. The PM_1_ sample from the birch log combustion reduced the amount of cells with intact cellular membranes to 60% and 25% at doses of 150 μg/ml and 200 μg/ml respectively (Fig 5A). The decrease of membrane integrity after exposure to the PM_1_ sample from the beech log combustion was not quite as pronounced with significance from unexposed control cells only at 200 μg/ml (Fig 5B). The most severe reduction of membrane integrity was found after exposure to the PM_1_ sample from the spruce log combustion (Fig 5C). We found no effects on cell membrane integrity from the PM_1_ sample from the pellet combustion (Fig 5D). There were significant differences between THP-1 and A549 cells at PM doses of 75 μg/ml and higher for the birch and spruce log combustion samples (Fig 5A and 5C); and 200 μg/ml for the beech log combustion sample (Fig 5B). The difference between the THP-1 cells and the co-cultured cells was significant at PM doses of 150 μg/ml and 200 μg/ml for the birch log combustion sample (Fig 5A) and at PM doses of 75 μg/ml and higher for the spruce log combustion sample (Fig 5C). No significant differences between THP-1 cells and the co-cultured cells were found for the beech log and pellet combustion samples (Fig 5B and 5D).

We could not detect a significant decrease of membrane integrity in A549 cells in the PI exclusion assay for any of the four tested PM_1_ samples (Fig 5A–5D).

In the co-culture, PM_1_ samples from the log wood combustions all caused minor dose-dependent decreases of cell membrane integrity, with significance from unexposed control cells at PM concentrations of 150 μg/ml and 200 μg/ml for all three samples (Fig 5A–5C). The PM_1_ sample from the pellet combustion did not cause significant decreases of membrane integrity in the co-cultured cells (Fig 5D). We did not find differences in the responses between A549 monocultures and the co-cultured cells for any tested sample (Fig 5A–5D).

### Genotoxicity

**Fig 6:**
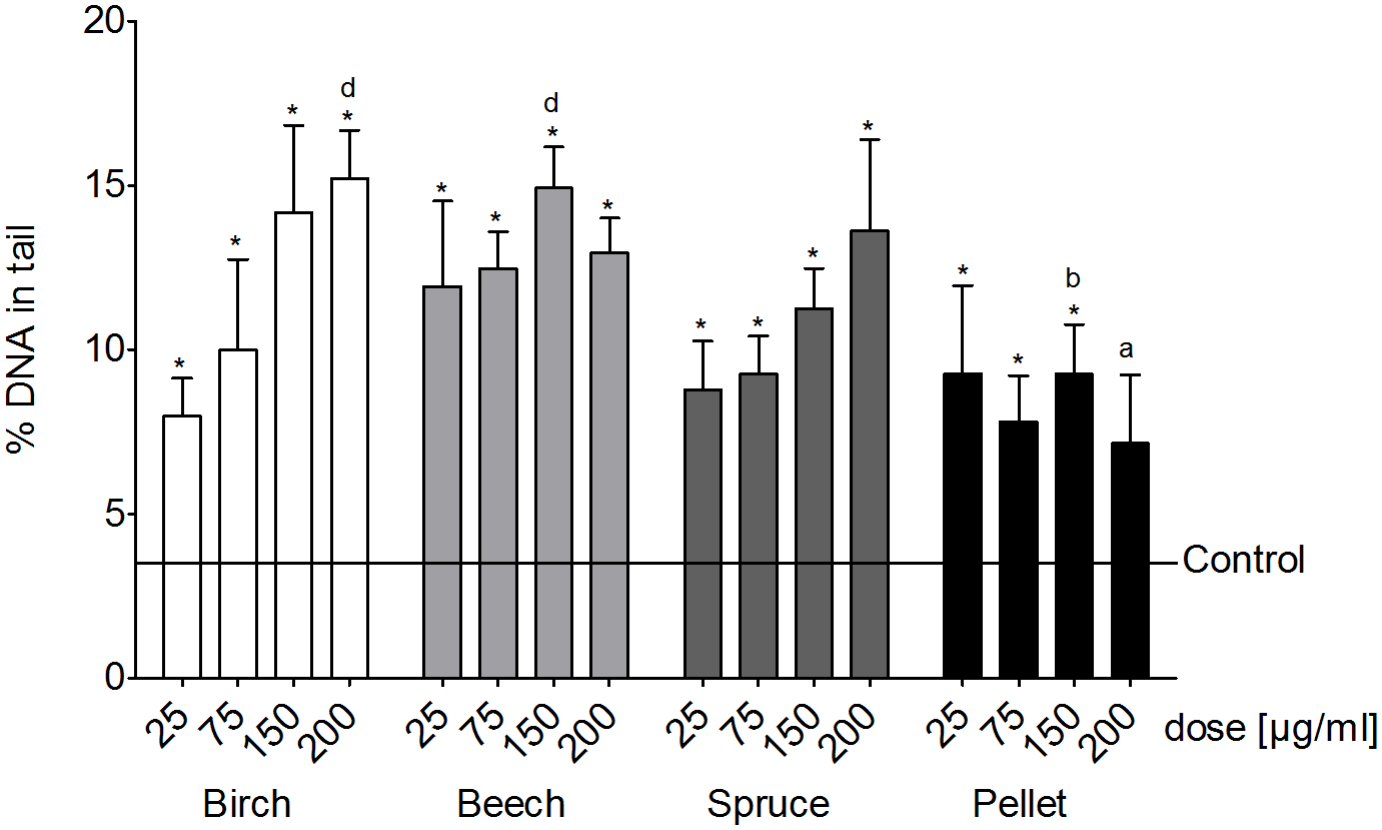
Genotoxicity. DNA fragmentation in THP-1 cells after a 24 h exposure to four doses (25, 75, 150 and 200 μg/ml) of PM_1_ samples from the combustion of three different wood logs and wood pellets expressed as percentage of DNA in the tail. Each bar represents the average of four independent experiments with 100 analyzed cells/assay + SEM of the experimental averages. Asterisks indicate statistical significance from blank control, **a** indicates significance from the birch log PM_1_ sample, **b** indicates significance from the beech log PM_1_ sample, **c** indicates significance from the spruce log PM_1_ sample, **d** indicates significance from the pellet combustion PM_1_ sample.

All of the samples caused an increase of the percentage of DNA in the tail in THP-1 cells (Fig 6). However, the pattern of the increase of DNA damage was divided. The PM_1_ samples from the birch and spruce log combustions followed dose-dependency, while there was no clear dose-dependency visible for the PM_1_ samples from the beech log combustion and the pellet combustion (Fig 6). Detailed results of the SCGE assay in A549 cells are described in detail in [18].

### Chemical composition of the PM samples and correlation with toxicity

The exact chemical composition of the PM_1_ samples is presented in [18]. Briefly, the log wood combustion PM_1_ samples contained significantly higher concentrations of OC and EC than the PM_1_ sample from the pellet combustion, and the OC/EC ration was the highest for the pellet combustion PM_1_ sample (S1 Table). Alkali and earth alkaline metals were generally found in much higher concentrations in the pellet combustion PM_1_ sample than in the log wood combustion samples, but the concentration of transition and heavy metals was on a similar scale in all four tested samples. The PAH content was higher in the log wood combustion samples than in the pellet combustion sample, however even between the log wood combustion samples the PAH content and composition varied considerably (S1 Table): the birch log combustion PM1 sample contained the overall lowest of PAH compounds, the PAH content of the PM1 sample from the spruce log combustion was more than three times higher. Especially the amount of alkylated and oxygenated PAH compounds was high in the PM1 sample from the spruce log combustion, with more than 20 times higher concentration than that of birch log combustion and approximately seven times higher than that of the beech log combustion.

Calcium was associated with a decrease in CMA and a decrease in membrane integrity in all three setups. Cadmium and organic compounds (PAH compounds, alkanes, OC and EC) were highly positively correlated with these two endpoints in THP-1 cells and co-cultured cells; however *ρ* for A549 cells was considerably smaller (Table 1). Generally speaking, the effects of the organic compounds on the cells’ metabolic activity and membrane integrity was far more pronounced in THP-1 and co-cultured cells than in A549 cells. We found positive correlations between the organic content of the samples and an increase in the concentration of pro-inflammatory cytokines (IL-8, IL-1β, TNFα), both in THP-1 monocultures and A549/THP-1 co-cultures; but the inorganic content of the samples was more highly associated with an increase in the GM-CSF concentration (Table 1). The decrease of TGFβ in the co-cultured cells was also correlated with an increased content or organics in the samples (Table 1). As for the A549 monocultures [18], also for THP-1 cells it was detected that the organic content of the samples was associated with increased DNA damage (Table 1).

**Table 1:** Spearman correlation of the chemical constituents with the toxicity endpoints.

	A549	THP-1	Co-culture	A549	THP-1	Co-culture	A549	THP-1	Co-culture	THP-1			Co-culture			
	Cellular Metabolic Activity			Membrane Integrity			Reactive Oxygen Species			DNA damage	IL-8	TNFα	TGFβ	GM-CSF	IL-1β	TNFα
Na	.109	-.404	-.159	-.327	-.271	.370	-.426	-.028	-.127	-.296	-.454	.021	.025	.768**	.039	.327
K	.297	-.263	.024	.056	-.102	.171	-.479	-.294	-.294	-.118	-.321	.076	-.232	.652**	-.032	.197
Rb	.262	-.230	.059	-.004	-.040	.231	-.464	-.302	-.299	-.111	-.266	.098	-.237	.682**	.022	.279
Ca	.632**	.561*	.746**	.365	.546*	.645**	.126	-.165	.226	.453	.450	.644**	-.906**	.493	.524*	.585*
Zn	-.068	-.025	.179	-.481	.209	.335	-.183	-.252	-.090	-.150	.150	.184	-.146	.714**	.457	.792**
Cd	.315	.863**	.944**	.042	.825**	.900**	.594*	.266	.490	.720**	.594*	.706*	-.825**	.708*	.706*	.818**
Pb	.540*	.253	.524*	.103	.322	.567*	-.083	-.146	.134	.211	.168	.540*	-.665**	.765**	.472	.684**
Cr	.268	.179	.434	-.124	.358	.532*	-.165	-.291	-.044	.053	.250	.394	-.509*	.789**	.518*	.791**
Mn	-.342	-.609*	-.390	-.701*	-.274	.002	-.790**	-.520	-.569	-.658*	-.356	-.303	.384	.584*	-.014	.399
Fe	.874**	.338	.641*	.587*	.368	.403	-.112	-.133	.119	.259	.287	.650*	-.790**	.427	.357	.420
Cu	.584*	.346	.536*	.077	.264	.712**	.248	.308	.500*	.338	.145	.705**	-.577*	.782**	.640**	.690**
Cl^-^	.203	-.212	.068	-.076	.016	.247	-.462	-.341	-.324	-.124	-.200	.082	-.218	.705**	.088	.368
SO_4_^2-^	.353	-.284	-.009	.124	-.157	.125	-.488	-.256	-.274	-.100	-.368	.071	-.218	.615*	-.071	.121
NO_3_^-^	.388	.307	.549*	.103	.447	.490	-.221	-.471	-.118	.106	.385	.438	-.688**	.609*	.450	.691**
Total PAH	.359	.905**	.885**	.371	.870**	.626**	.521*	-.082	.318	.650**	.900**	.600*	-.859**	-.082	.582*	.453
Genotoxic PAH (TEQ)	.312	.702**	.749**	.388	.779**	.356	.276	-.385	.032	.459	.924**	.571*	-.771**	-.084	.429	.21
Alkylated PAH	.301	.767**	.752**	.273	.820**	.380	-.112	-.288	.103	.465	.876**	.426	-.709**	-.222	.426	.365
Oxygenated PAH	.376	.928**	.906**	.362	.876**	.664**	.579*	-.009	.385	.709**	.885**	.626**	-.874**	-.031	.621*	.476
Other PAH	.415	.914**	.920**	.376	.806**	.685**	.621*	.182	.512*	.824**	.779**	.641**	-.888**	.018	.568*	.394
alkanes	.441	.878**	.926**	.159	.824**	.872**	.591*	.194	.574*	.668**	.800**	.797**	-.853**	.347	.859**	.771**
OC	.902**	.747**	.925**	.559	.736**	.778**	.**413**	.385	.601*	.601*	.734**	.937**	-.965**	.361	.699*	.664*
EC	.741**	.925**	.963**	.413	.869**	.820**	.776**	.734**	.902**	.748**	.930**	.937**	-.895**	.182	.825**	.685*

Spearman correlation coefficients (ρ) between the chemical constituents of the particulate samples from the combustion of different wood logs and wood pellets and the toxicological responses of the three cell culture setups (A549 and THP-1 monocultures, and A549/THP-1 co-culture). Boldfaced values indicate statistical significance:

**p < 0.01,

*p < 0.05

## Discussion

### Chemical composition of the tested particles

The masonry heater and the pellet boiler used as emission sources in the present and a previous [18] study have been chosen to represent two prevalent domestic biomass heating systems. Nonetheless, their combustion quality and combustion conditions vary significantly, which greatly influences the chemical composition of their PM emissions [25,26]. Generally, the PM_1_ emissions from the three log wood combustion contained higher amounts of PAH compounds, which is an indication of incomplete combustion and lower combustion temperatures, while the PM_1_ sample from the pellet combustion contained high amounts of inorganic salts. In previous studies [27,28,29] these inorganic salts were associated only with low toxic effects *in vitro* and *in vivo*. In contrast, transition and heavy metals have previously been associated with a high inflammatory potential, as well as high cytotoxicity [29]. However, these previous studies did not conduct a systematic comparison between the responses of epithelial and immune cells, the two cell types, which are most likely to interact with the particles upon inhalation. Nor, did they elucidate the effect on cell-cell interaction on the extent of PM toxicity.

### Oxidative stress and inflammatory responses

In contrast to previous studies with A549 monocultures [18,30], the same significant increase of cellular ROS production was not seen in the THP-1 monoculture nor the A549/THP-1 co-culture. This indicates mitigating effects of the macrophages on the oxidative stress caused by the exposure of the particulate samples. A previous study [31], found that alveolar macrophages protected A549 cells from oxidative damage and attributed this to the macrophages’ ability to effectively sequester Fe-ions from the medium and thus minimize Fe-driven (Fenton-type) oxidative reactions. However, the use of mouse macrophages in combination with human alveolar cells probably does not reflect the actually occurring cell-cell interactions in the human lung. Studies, which systematically compare the oxidative stress effects of the same particulate samples in mono- and co-cultured respiratory cells are rare, while inflammatory responses are slightly better understood. Roqué et al. [32] and Hofmann et al. [33] found that immune cells are the primary mediators of neuroinflammation [32] or inflammatory responses in murine alveolar epithelial cells or murine precision-cut lung slices [33]. Further studies are needed to elucidate the exact molecular mechanisms by which a relatively small amount of macrophages can protect alveolar epithelial cells from oxidative damage.

One major physiological function of TNFα *in vivo* is the activation of nuclear factor kappa-light-chain-enhancer of activated B cells (NF-κB) and the induction of CXCL-1 mediated influx of neutrophils to the site of inflammation [34]. While TNFα was not increased after exposure of the A549 cells to any of the PM_1_ samples, we did find an increase in TNFα production for all four tested samples both in the THP-1 monoculture and the co-culture of A549 and THP-1 cells. In a co-culture study, conducted with murine alveolar epithelial cells (MLE-12) and macrophages (RAW264.7), a similar trend in TNFα production was found after the exposure of the cells to the urban reference particles SRM 1648 [35]. The authors found no increased production of TNFα in the epithelial cells, however, TNFα production was increased in macrophage monocultures and MLE-12/RAW264.7 co-cultures. Similarly to the present results, also Musah et al. [35] found that the absolute concentration of TNFα was significantly higher in monocultures of the macrophages than in the co-cultures, further corroborating that TNFα is indeed primarily secreted by macrophages. As pointed out by Chen et al. [36], it is the CXCL-1 mediated influx of neutrophils which is the hallmark of acute lung inflammation *in vivo*. The authors concluded that CXCL-1 is mainly secreted by the alveolar epithelial cells, not the alveolar macrophages. TNFα, which is needed to trigger CLCX-1 mediated neutrophilic influx, is not secreted by alveolar macrophages, while CLCX-1 is not secreted by macrophages. This further underlines the need to incorporate fast and easily reproducible *in vitro* co-culture exposures into regular PM toxicity testing. Our correlation analysis showed that in the co-cultured cells mainly metals (Cd, Cr, Cu, Ni, Pb and Zn) and alkanes were strongly positively associated with an increase in TNFα secretion. This result concurs with previous findings [37,38,39], which have found that it is mainly the metal content of the PM, which is responsible for inflammatory effects.

Overall, the inflammatory responses found in this study were low compared to exposure studies with ambient air particles [37,40]. In A549 cells we found only an increase in the production of IL-8 [18] while the other measured cytokines were below detection level. The absolute concentration of IL-8 by THP-1 cells after exposure to the PM_1_ samples was approximately the same as that of the A549 cells, but the basal level of IL-8 in THP-1 cells was lower, thus the relative increase by THP-1 cells was higher than that of the A549 cells. Contrastingly, in the co-cultured-cells, the basal level of IL-8 was already approximately 47 ng/ml, compared to 100 pg/ml in THP-1 cells and 230 pg/ml for A549 cells. The relative increase in the production of IL-8 however, was smaller in the co-cultured cells than in either monoculture.

In our previous study [18] we concluded that the PM_1_ sample from the pellet combustion does not cause inflammation, however, now we showed that for some pro-inflammatory parameters, e.g. GM-CSF, the PM_1_ sample from the pellet combustion was the only sample to induce any measurable response. The main function of GM-CSF in the airways is to stimulate proliferation of alveolar macrophages and type II alveolar epithelial cells and induce transcription factor PU.1 [41], however overexpression of GM-CSF has been associated with pulmonary fibrosis [42]. Our correlation analysis showed that mostly ions (Na, K, and Cl) and metals (Cr, Cu, and Pb) were positively associated with an increase in GM-CSF production. This further strengthens previous findings that it is mainly the metal content of combustion-derived PM, which induces inflammatory reactions both *in vitro* and *in vivo* [29,39].

Interestingly, while Xing et al. [42] have concluded that an over-expression of GM-CSF induces induction of TGFβ, we found that TGFβ levels were decreased in a dose-dependent manner by all the tested PM_1_ samples. A major signaling function of TGFβ is the induction of apoptosis through either SMAD-mediated induction of mitogen-activated protein kinase (MAPK) pathways. However, the exact signaling pathways induced by TGFβ vary greatly depending on the effector and signaling cells. For example, it has been shown that TGFβ can inhibit NF-κB activation [43], thus a reduction of TGFβ as seen in our study could lead to further activation of NF-κB signaling, which, in combination with the increase in TNFα secretion we have seen, could lead to a prolonged activation of NF-κB, which could eventually lead to the formation of tumors.

### Cell viability

Regarding the viability of the cultured cells, we found a similar trend as in our previous study [18], meaning that the PM_1_ sample from the pellet combustion had the least effects on cell viability. This was evident in both the THP-1 monoculture and the A549/THP-1 co-culture. This sample is composed mainly of inorganic components, such as sodium, potassium and sulfate [18], all of which have been shown to cause very little toxicity in previous studies [26,27]. All of the log wood combustion samples induced a far greater reduction of the cellular metabolic activity (CMA) in both the THP-1 mono- and the A549/THP-1 co-cultures. Correlation analysis revealed that carbonaceous components, especially OC and EC, had the highest positive associations with a reduction of the CMA in all three setups.

Interestingly, the CMA of the co-cultured cells was on the same level as the CMA of the THP-1 monoculture, even though the percentage of THP-1 cells was only between 5–10%. This indicates the involvement of intercellular communication between the THP-1 and the A549 cells and an influence on the viability of the other cell types. Hence, even if particles are not harmful to only epithelial cells, they still might cause cell damage in alveoli. For our second marker of cell viability, the membrane permeability of the cells, the response of the co-cultured cells was more in line with the A549 monoculture. Thus, we can postulate that the activated macrophages lead to an increased reduction of the CMA of epithelial cells, which might eventually lead to apoptotic cell death.

### Genotoxicity

In contrast to our previous findings in the A549 cell line [18], where only the PM_1_ sample from the beech and spruce log wood combustion caused a significant increase in the amount of DNA fragmentation, in THP-1 cells all PM_1_ samples caused a significant increase in DNA fragmentation. However, a clear dose-dependency was found only for the birch and spruce combustion samples and not for the beech and pellet combustion samples. Correlation analysis showed that in A549 cells Fe, Ca and NO_3_^-^ and PAH compounds had strong positive associations with an increase of the percentage of DNA in the tail, in addition to PAH compounds [18]. Contrastingly, for THP-1 cells we did not find the same correlation between the Fe and Ca content of the samples and DNA damage. Rather, EC and OC had high positive correlations in addition to the PAH compounds. It would have been of high interest to test the potential of the PM_1_ sample to damage DNA also in the co-culture setup, however the amount of sample was limited and thus the SCGE-assay could not be conducted for the co-cultures. From our correlation analysis, it is clear that PAH compounds are associated with an increase in DNA damage in both monocultures. Thus, it is safe enough to assume that most of the genotoxic effects from these samples stem from DNA-PAH-adduct formation [44] and only to a lower extent from the effects of sustained inflammatory reactions and consequent NF-κB activation as reviewed by Pal et al. [45].

## Conclusion

We could show that the magnitude of toxicological responses varies significantly between different *in vitro* setups, thus testing the toxicity of combustion-derived PM in only one cell line can under- or overestimate its induced adverse health effects. While the PM_1_ sample from the pellet combustion did not induce significant cell death or inflammation in the monoculture of alveolar epithelial cells, in macrophages and a co-culture of epithelial cells and macrophages, this sample caused significant increases in important pro-inflammatory markers, such as TNFα and GM-CSF. Of the log wood combustion samples only the birch log combustion sample exhibited inflammatory responses in A549 epithelial cells, while in the THP-1 monoculture and the co-culture of A549 and THP-1 cells also the other two log wood combustion samples caused significant increases of pro-inflammatory markers. It seems that the chemical composition of the pellet combustion PM_1_ sample does not affect epithelial cells as much as it does the other two cell culture setups. Also, transition and heavy metals in the samples, affect mainly immune cells and the co-culture of immune and epithelial cells. All in all, it is clear from this study, that to completely evaluate, which of the complex pro-inflammatory cascades are induced by PM, more than one *in vitro* exposure setup should be used.

Conclusively, our results support that OC, EC and PAH compounds are associated with the observed cytotoxicity in all three experimental setups, while the metal content correlates with the inflammatory potential of the samples. Furthermore, our results suggest that the content of PAH compounds in the PM samples is the main cause of DNA-damage. Thus, the physicochemical composition of the sample determines which pathway of toxicity is induced.

Finally, it is clear from this and our previous study, that all of the tested wood combustion-derived PM samples induce adverse effects and that rather than preferring one type of fuel or combustion appliance over the other, the main effort should be put into minimizing PM emissions. The results obtained from these and future *in vitro* exposure experiments will need to be evaluated against results from *in vivo* studies to find the most suitable *in vitro* setups and endpoints and to use *in vitro* toxicity screening in favor of *in vivo* screening where applicable.

## Supporting information

S1 Fig TNFα production by THP-1 cells. Production of the pro-inflammatory marker TNFα after a 24 h exposure of THP-1 cells to four doses (25, 75, 150 and 200 μg/ml) of PM_1_ samples from the combustion of three different wood logs and wood pellets. Bars represent the increase in TNFα secretion by the cells in comparison to unexposed control cells + SEM of the experimental averages. Asterisks indicate significance from blank control.
(TIF)

S2 Fig Production of pro-inflammatory markers of am A549/THP-1 co-culture. Production of the pro-inflammatory markers IL-10, VEGF-A, IL-6 and IL-8 by a co-culture of A549 and THP-1 cells after a 24 h exposure of the cells to four doses (25, 75, 150 and 200 μg/ml) of PM_1_ samples from the combustion of three different wood logs and wood pellets. Bars represent the fold-change compared to unexposed control cells + SEM of the experimental averages. Asterisks indicate significance from unexposed control cells.
(TIF)

S1 Table PAH and alkane concentration in the PM_1_ samples. Concentration of polycyclic aromatic hydrocarbons and alkanes in the PM_1_ emissions from the combustion of three types of wood logs (birch, beech and spruce) and spruce pellets. Concentrations are provided in ng/mg sample mass. bdl = below detection limit.
(PDF)

## Abbreviations

ANOVA: analysis of variance
ARDS: adult respiratory distress syndrome
AUC: area under the curve
CMA: cellular metabolic activity
DCF: 2’, 7’–dichlorofluorescein
DMEM: Dulbecco’s Modifies Eagle Medium
DSMZ: German Collection of Microorganisms and cell cultures
EC: elemental carbon
EDTA: Ethylenediaminetetraacetic acid
ELISA: enzyme-linked immunosorbent assay
EPA: US Environmental Protection Agency
FBS: fetal bovine serum
GM-CSF: granulocyte macrophage colony-stimulating factor
H_2_DCF-DA: 2’,7’–dichlorofluorescin diacetate
IARC: International Agency for Research on Cancer
IFNγ: interferon gamma
IL-1: interleukin 1 beta
IL-10: interleukin 10
IL-6: interleukin 6
IL-8: interleukin 8
L-glut: L-glutamine
MAPK: mitogen-activated protein kinase
MTT: 3-(4,5-dimethylthiazol-2-yl)-2,5-diphenyltetrazolium bromide
NF-κB: nuclear factor kappa-light-chain-enhancer of activated B cells
OC: organic carbon
PAH: polycyclic aromatic hydrocarbon
PBS: Dulbecco’s phosphate buffered saline
PI: propidium iodide
PM: particulate matter
PMA: phorbol 12-myristate 13-acetate
ROS: reactive oxygen species
RPMI: Roswell Park Memorial Medium
SCGE: singe cell gel electrophoresis
SDS: sodium dodecyl sulfate
TGFβ: transforming growth factor beta
TNFα: tumor necrosis factor alpha
