# Supplementary figures and images for: Differences between co-cultures and monocultures in testing the toxicity of particulate matter derived from log wood and pellet combustion
Source: PLoS One. 2018 Feb 21;13(2):e0192453. doi: 10.1371/journal.pone.0192453 (PMC5821343; doi:10.1371/journal.pone.0192453)

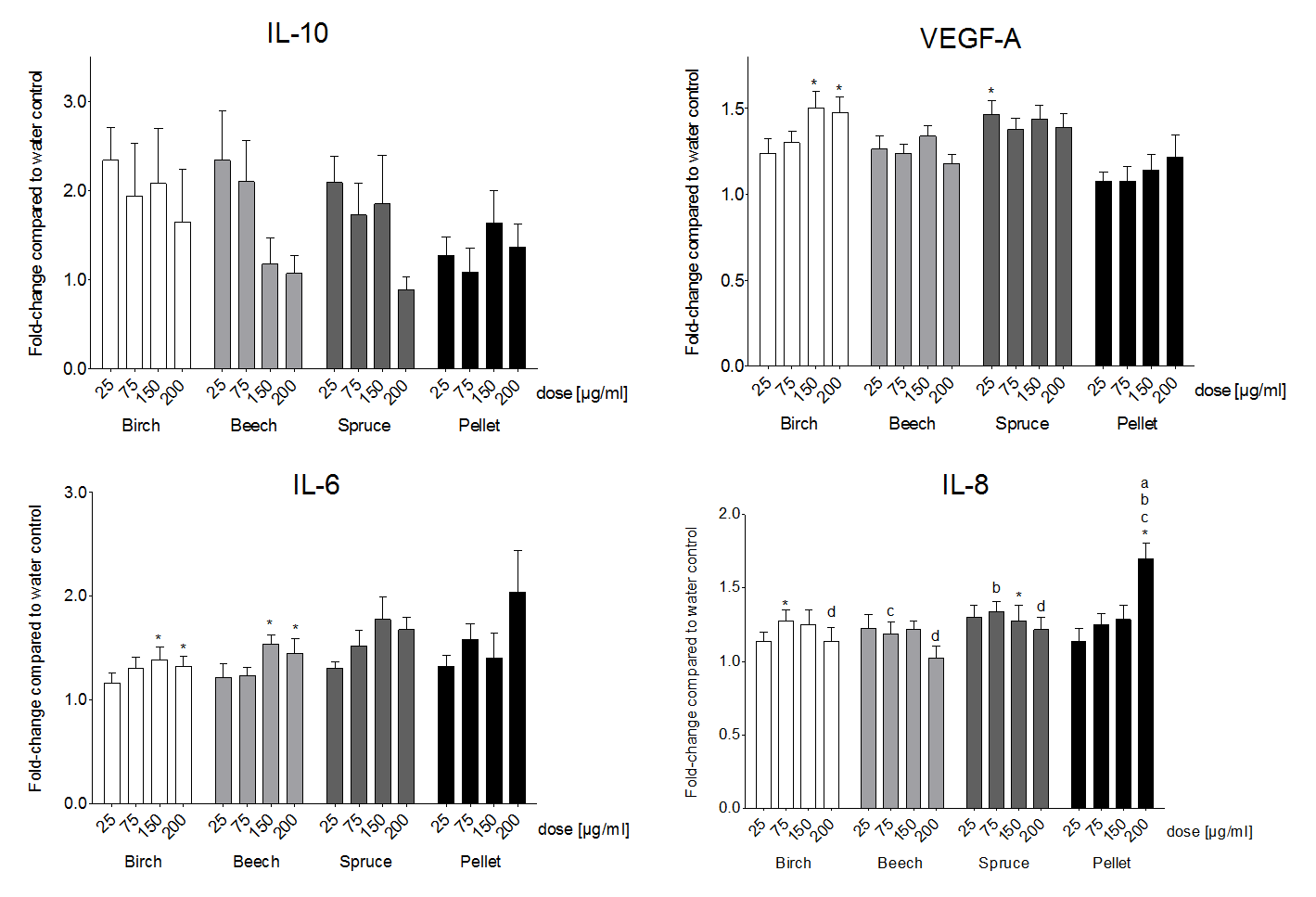

Supplement: S2 Fig — Production of the pro-inflammatory markers IL-10, VEGF-A, IL-6 and IL-8 by a co-culture of A549 and THP-1 cells after a 24 h exposure of the cells to four doses (25, 75, 150 and 200 μg/ml) of PM1 samples from the combustion of three different wood logs and wood pellets. Bars represent the fold-change compared to unexposed control cells + SEM of the experimental averages. Asterisks indicate significance from unexposed control cells. (TIF) [file pone.0192453.s002.tif]
