# Supplementary material for: Differences between co-cultures and monocultures in testing the toxicity of particulate matter derived from log wood and pellet combustion
Source: PLoS One. 2018 Feb 21;13(2):e0192453. doi: 10.1371/journal.pone.0192453 (PMC5821343; doi:10.1371/journal.pone.0192453)
Supplement: S1 Table — Concentration of polycyclic aromatic hydrocarbons and alkanes in the PM1 emissions from the combustion of three types of wood logs (birch, beech and spruce) and spruce pellets. Concentrations are provided in ng/mg sample mass. bdl = below detection limit. (PDF) [file pone.0192453.s003.pdf]

	Birch	Beech	Spruce	Pellet
Naphthalene	2.1	bdl	0.3	0.2
Acenaphthylene	4.3	2.7	0.5	bdl
Acenaphthene	bdl	bdl	bdl	bdl
Fluorene	5.8	5.6	1.6	bdl
Phenanthrene	156.9	151.9	77.6	1.4
Anthracene	7.7	14.6	8.2	bdl
Pyrene	56.9	125.4	174.1	6.0
1-Methylphenanthrene	12.9	9.3	22.4	0.1
Fluoranthene	75.9	145.8	200.9	5.2
Benzo[c]phenanthrene	5.5	13.9	31.9	1.1
Benzo[a]anthracene	4.3	16.9	87.9	1.6
Cyclopenta[c,d]pyrene	2.0	14.1	39.4	1.3
Triphenylene	2.8	9.2	24.5	bdl
Chrysene	7.4	17.7	80.0	2.7
5-Methylchrysene	bdl	0.4	0.8	bdl
Benzo[b]fluoranthene	5.3	25.4	101.9	1.0
Benzo[k]fluoranthene	bdl	bdl	bdl	0.2
Benzo[j]fluoranthene	3.8	20.0	67.0	0.7
Benzo[e]pyrene	4.9	21.1	62.1	1.1
Benzo[a]pyrene	2.3	18.7	82.4	0.4
Perylene	0.6	3.5	13.1	bdl
Indeno[1,2,3-cd]pyrene	0.4	5.1	58.5	bdl
Dibenzo[a,h]anthracene	bdl	bdl	9.5	bdl
Benzo[g,h,i]perylene	1.0	7.8	63.4	0.3
Anthanthrene	bdl	bdl	14.4	bdl
Dibenzo[a,l]pyrene	bdl	bdl	1.1	bdl
Dibenzo[a,e]pyrene	bdl	bdl	7.0	1.4
Coronene	bdl	bdl	30.8	bdl
Dibenzo[a,i]pyrene	bdl	bdl	bdl	bdl
Dibenzo[a,h]pyrene	bdl	bdl	bdl	bdl
2-/8-Methylfluoranthene	bd	2.9	9.8	bdl
1-/3-/7-Methylfluoranthene	1.6	6.0	17.1	bdl
4-Methylpyrene	bdl	2.2	7.4	bdl
2-Methylpyrene	0.9	2.9	8.7	bdl
1-Methylpyrene	0.8	2.9	9.1	bdl
7,12-Dimethyl-Benz[a]anthracene	4.2	4.8	bdl	bdl
9-Methylphenanthrene	3.7	4.4	2.4	bdl
2,6-Dimethoxybenzoquinone	37.3	83.5	bdl	bdl
9H-Fluoren-9-one	47.0	44.2	13.3	bdl
1H-Phenalen-1-one	29.9	47.2	61.7	0.6
Xanthone	5.4	5.5	4.4	bdl
9,10-Anthracenedione	15.2	17.6	26.6	bdl
Cyclopenta(def)phenanthrenone	19.3	28.3	31.0	0.7
1,8-Naphthalic anhydride	58.0	158.0	246.0	bdl
2,3-5,6-Dibenzoxalene	2.3	4.3	11.4	bdl

Benzo[b]naphtho[2,3-d]furan	1.0	2.3	9.7	bdl
Benzo[kl]xanthene	1.2	3.9	11.5	bdl
11H-Benzo[a]fluoren-11-one	1.8	4.1	17.1	bdl
4-Oxapyrene-5-one	3.6	5.3	11.0	ndl
7H-Benzo[c]fluorene-7-one	1.1	2.1	7.7	bdl
11H-Benzo[b]fluoren-11-one	1.8	5.0	21.6	bdl
7H-Benz[de]anthracen-7-one	3.9	11.6	43.1	bdl
Naphtho[2,1,8,7-klmn]xanthene	bdl	1.2	3.2	bdl
Benz[a]anthracene-7,12-dione	bdl	1.2	5.5	bdl
5,12-Naphthacenedione	bdl	bdl	2.5	bdl
Phenanthro[3,4-c]furan-1,3-dione	bdl	2.1	8.7	bdl
6H-Benzo[cd]pyren-6-on	3.0	16.3	60.3	bdl
Tetradecane	3.5	bdl	bdl	bdl
Pentadecane	bdl	bdl	bdl	bdl
Hexadecane	9.7	7.1	bdl	bdl
Heptadecane	10.9	8.0	1.5	bdl
Octadecane	8.8	6.6	bdl	bdl
Nonadecane	5.5	5.5	2.9	1.5
Eicosane	13.7	8.1	12.7	4.0
Heneicosane	3.2	4.0	5.4	1.2
Docosane	8.1	4.6	10.7	4.9
Tricosane	1.5	4.5	11.6	1.8
Tetracosane	5.1	4.9	10.8	3.0
Pentacosane	0.9	bdl	5.7	1.2
Hexacosane	3.2	1.1	6.8	1.4
Heptacosane	3.0	bdl	5.4	3.8
Octacosane	2.4	1.4	5.3	1.7
Nonacosane	7.3	1.1	3.2	4.3
Triacontane	bdl	0.8	2.2	1.0
Hentriacontane	bdl	0.3	0.6	0.7
Dotriacontane	bdl	bdl	0.8	0.4
Tritriacontane	3.2	bdl	2.4	1.6
